# Implications of the first recording of European silver eels *Anguilla anguilla* in the inland waters of Cyprus

**DOI:** 10.1111/jfb.70357

**Published:** 2026-02-10

**Authors:** Sotiris Meletiou, Demetra Andreou, Rosalind M. Wright, J. Robert Britton, Nathan P. Griffiths, Marlen I. Vasquez

**Affiliations:** ^1^ Department of Life and Environmental Sciences, Faculty of Science and Technology Bournemouth University Poole UK; ^2^ Department of Chemical Engineering, Faculty of Geotechnical Sciences and Environmental Management Cyprus University of Technology Limassol Cyprus; ^3^ Environment Agency Feering UK; ^4^ Institute for Biodiversity and Freshwater Conservation University of the Highlands and Islands Inverness UK

**Keywords:** barriers to migration, catadromous, eel management plans, escapement, Mediterranean

## Abstract

Migrating silver European eels *Anguilla anguilla* are reported from the inland waters of the island of Cyprus for the first time, expanding the known geographic range in the Eastern Mediterranean of emigrating adults of this critically endangered, catadromous species. Silver eels were captured attempting to migrate to sea from two separate locations. This is important, as Cyprus was previously exempt from European eel management plans due to a presumed absence of significant eel populations, especially of the silver life stage. This now requires urgent review to ensure these populations are managed appropriately.

The European eel *Anguilla anguilla* L. (“eel”) is a catadromous species with a lifecycle involving migrations between their spawning areas in the Sargasso Sea and European freshwater and coastal habitats (Righton et al., 2016; Wright et al., 2022). Juvenile eels use these European habitats for development until they approach sexual maturity, when they metamorphose into the silver eel stage and prepare to migrate to their spawning areas (Schmidt & Regan, 1923). European eel populations have dramatically declined over the past century (Correia et al., 2018), with current estimates indicating that recruitment has fallen to less than 10% of historical levels (O'Leary et al., 2022). Multiple factors are suggested as contributing to eel population declines, including habitat fragmentation through barrier construction (Nilsson et al., 2005), pollution (Pujolar et al., 2013), extreme weather conditions (Clark et al., 2002), parasite infections (Lefebvre et al., 2013) and exploitation (Dekker, 2003). However, issues associated with the marine environment (Wright et al., 2022) and silver eel escapement into inshore areas from riverine environments (Höhne et al., 2023) are recognised as key factors in the population decline. European eel populations in the Mediterranean Sea have recently seen the lowest recruitment levels ever recorded, with a scientific assessment suggesting no overall progress in the recovery of their populations (Ciccotti & Morello, 2023).

The decline of the eel population has resulted in their assessment as “Critically Endangered” on the IUCN Red List since 2008 (Pike et al., 2020). European Union legislation (EC No 1100/2007) has mandated the development of Eel Management Plans (EMPs) to help conserve their remaining populations. These plans aim to protect and promote the recovery of European eel populations, including achieving silver eel escapement to the spawning stock that is equal to or exceeds 40% of the potential biomass expected in the absence of anthropogenic disturbance and exploitation. Some European countries or regions where there are no records or the environment is not considered suitable to support eel populations have been exempted from developing EMPs, such as the Mediterranean island of Cyprus.

As the eel populations around the Mediterranean Sea remain imperilled, with no overall progress in their recovery (Ciccotti & Morello, 2023), this discovery of all eel life stages of an unexploited eel population in Cyprus provides an opportunity for this island to contribute to the recovery of eel populations in the region. This is the first step in the development and implementation of the EMP, which we suggest is required urgently.

Cyprus is characterised by freshwater habitats that are prone to drying in summer, with water shortages resulting in freshwater resource management practices that aim to ensure the provision of water to people and industry all year round, such as through dam and reservoir construction (Rocha et al., 2020). The historical exemption of Cyprus from implementing EMPs was initially challenged by results of fish surveys conducted by electric fishing (Zogaris et al., 2012). Fyke netting and environmental DNA have also recently confirmed the presence of eel populations in the island's freshwaters (Griffiths et al., 2023). Glass eel sampling in refuge traps confirmed recruitment occurs in March, with elver and yellow eel distribution restricted to areas of low elevation (data not shown). There was a negative association between eel presence and distance from the coast and barrier presence (Griffiths et al., 2023; Zogaris et al., 2012). No evidence was recorded of these eels metamorphosing to the silver eel stage. The lack of evidence that eels survive in the highly fragmented and ephemeral rivers long enough to mature and migrate as silver eels has meant Cyprus has continued to be exempt from developing and implementing EMPs.

This apparent absence of silver eels in inland waters of Cyprus can now be challenged directly following surveys completed at Oroklini lake (34.970731, 33.654347) in December 2024 and Polis River (35.031902, 32.424179) in February 2025 (Figure 1). Two fyke nets (1.0 × 2.3 m) were deployed overnight in Oroklini Lake, a Natura 2000 protected site, between 2‐4 December 2024. For the Polis River, electric fishing surveys using back‐mounted SmithRoot LR24 equipment took place between 24 February 2025 and 1 March 2025. All samplings were completed under permits and licences obtained from the competent authorities of Cyprus (Water Development Department) (02.15.002), Department of Fisheries and Marine Research (04.02.015.008) and the Environment Department (02.15.002.004). The captured silver eels were measured (total length, nearest mm) and weighed (nearest g). The environmental parameters of water temperature, dissolved oxygen, pH and conductivity were recorded at the time of sampling using a portable multimeter (Hanna instruments HI‐98194, USA).

**FIGURE 1:**
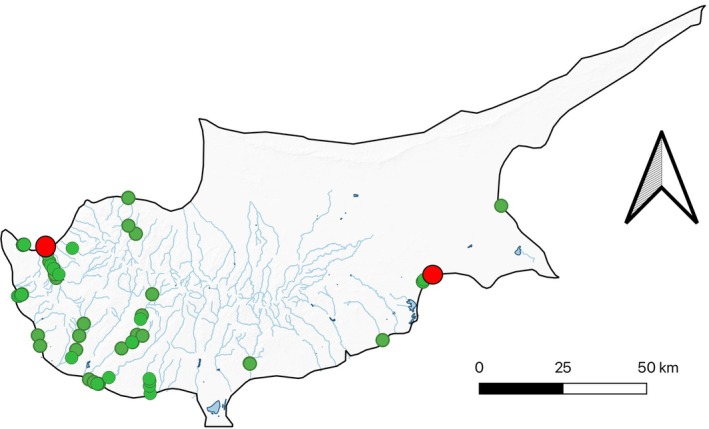
Distribution of European eel (*Anguilla anguilla*) records in Cyprus. Green symbols indicate sites with historical or recent eel presence based on electrofishing and environmental DNA surveys, while red symbols mark the first confirmed inland records of silver eels (current study).

Water level records from the Water Development Department of Cyprus indicated that on 3 December 2024, Oroklini Lake's water level remained below the critical threshold of 1.0 m that meant the barrier at the outflow of the lake prevented water egress. On the following day, heavy rainfall caused the lake to overflow over the lake's weir, resulting in the fyke nets capturing 473 silver eels. As construction work meant natural connectivity between the Oroklini Lake (34.970731, 33.654347) and the sea had been lost, the silver eels were transported to the sea following a conditioning phase to facilitate their emigration. This phase involved a short period of rest (30 min) and gradual salinity acclimation with stepwise increases from freshwater to brackish and then full‐strength seawater under controlled temperature and high dissolved oxygen to minimise handling and osmotic stress prior to release.

Water quality parameters recorded during the sampling in Oroklini Lake were pH 8.29, dissolved oxygen at 158.3% saturation, ambient conductivity of 5525 μS/cm, and a water temperature of 16.3°C. For the eels sampled, total length ranged from 382 to 680 mm (mean ± standard deviation 456 ± 60 mm) and body mass from 120 to 555 g (mean 173 ± 83 g). Key morphometric measurements were recorded to confirm the silvering stage of the captured eels (Table 1), with the horizontal and vertical eye diameters notably enlarged, a known indicator of the silver eel stage (Pankhurst & Lythgoe, 1982). Additionally, observed pigmentation characteristics and body morphology were consistent with criteria established by Durif et al. (2005) for silver eel classification.

**TABLE 1:** Morphometric characteristics and silvering classification of European eels (*Anguilla anguilla*) captured in Cyprus.

No	Eel ID	Capture date	Total length (cm)	Weight (g)	Head length (mm)	Horizontal eye diameter (mm)	Vertical eye diameter (mm)	Pectoral fin length (mm)	Durif silvering stage	Eye index	Pectoral fin index
1	ORO‐F1‐3	4 December 2024	55	295	59	7.5	7.3	37	IV/V (silver)	0.125	0.627
2	ORO‐F1‐4	4 December 2024	49	205	55.4	5.6	7.3	28	IV/V (silver)	0.116	0.505
3	ORO‐F1‐5	4 December 2024	59.5	350	68.1	6	8.3	33.1	IV/V (silver)	0.105	0.486
4	ORO‐F1‐6	4 December 2024	62	380	75.5	6.7	6.9	32.8	IV/V (silver)	0.090	0.434
5	ORO‐F1‐7	4 December 2024	49	205	54.3	6.2	6.2	27.6	IV/V (silver)	0.114	0.508
6	ORO‐F1‐8	4 December 2024	60.5	380	69.1	8.6	8.7	45.4	IV/V (silver)	0.125	0.657
7	ORO‐F1‐9	4 December 2024	54	260	54.3	7.3	7.7	29	IV/V (silver)	0.138	0.534
8	ORO‐F1‐10	4 December 2024	43	150	46.2	7.2	6.4	26.2	IV/V (silver)	0.147	0.567
9	ORO‐F1‐11	4 December 2024	51.8	150	57.5	5.6	7.5	33	IV/V (silver)	0.114	0.574
10	ORO‐F1‐12	4 December 2024	42	175	46.5	6.8	7.1	25.3	IV/V (silver)	0.149	0.544
11	ORO‐F1‐13	4 December 2024	50.1	240	55	5.9	6	26.6	IV/V (silver)	0.108	0.484
12	ORO‐F1‐14	4 December 2024	41	120	49.1	8.6	7.6	23.9	IV/V (silver)	0.165	0.487
13	ORO‐F1‐15	4 December 2024	39	130	51.2	6	7.8	24.8	IV/V (silver)	0.135	0.484
14	ORO‐F1‐16	4 December 2024	41	175	43.1	7.6	8.1	24.8	IV/V (silver)	0.182	0.575
15	ORO‐F1‐17	4 December 2024	46.8	160	51.6	7.9	8.4	29.5	IV/V (silver)	0.158	0.572
16	ORO‐F1‐18	4 December 2024	39.5	120	46.3	6.6	6.3	26.4	IV/V (silver)	0.139	0.570
17	ORO‐F1‐19	4 December 2024	68	555	84.3	6.7	5.2	44.1	IV/V (silver)	0.071	0.523
18	ORO‐F1‐20	4 December 2024	50.9	275	45.4	6.2	5.7	30.9	IV/V (silver)	0.131	0.681
19	ORO‐F1‐21	4 December 2024	43.1	170	45	6.9	7.2	27.8	IV/V (silver)	0.157	0.618
20	ORO‐F1‐22	4 December 2024	41.4	130	38.3	7	6.9	26.4	IV/V (silver)	0.181	0.689
21	ORO‐F1‐23	4 December 2024	55.8	375	56.5	5.5	4.9	30.1	IV/V (silver)	0.092	0.533
22	ORO‐F1‐24	4 December 2024	41	155	44.3	6.4	7.2	20.8	IV/V (silver)	0.153	0.470
23	ORO‐F1‐25	4 December 2024	39.1	150	39.8	6.3	5.8	24.2	IV/V (silver)	0.152	0.608
24	ORO‐F1‐26	4 December 2024	46.2	195	48	5.6	6.9	29.1	IV/V (silver)	0.130	0.606
25	ORO‐F1‐27	4 December 2024	43	155	40	5.4	6.5	24.4	IV/V (silver)	0.149	0.610
26	ORO‐F1‐28	4 December 2024	44.5	190	50.2	6.8	7.6	28.6	IV/V (silver)	0.143	0.570
27	ORO‐F1‐29	4 December 2024	45.2	245	57.8	5.1	6.2	30.2	IV/V (silver)	0.098	0.522
28	ORO‐F1‐30	4 December 2024	42	155	46.9	5.8	6.9	25	IV/V (silver)	0.135	0.533
29	ORO‐F1‐31	4 December 2024	38.5	160	43.8	6.3	6.7	23.5	IV/V (silver)	0.148	0.537
30	ORO‐F1‐32	4 December 2024	45.8	170	43.5	5.2	6.5	22.5	IV/V (silver)	0.134	0.517
31	ORO‐F1‐33	4 December 2024	39.9	140	46	7	8.1	22.2	IV/V (silver)	0.164	0.483
32	ORO‐F1‐35	4 December 2024	40.4	125	42.4	6.5	5.8	24.3	IV/V (silver)	0.145	0.573
33	ORO‐F1‐36	4 December 2024	39.4	150	46.9	5.5	5.8	22.3	IV/V (silver)	0.120	0.475
34	ORO‐F1‐37	4 December 2024	40.8	165	42.5	5.5	5.7	25.1	IV/V (silver)	0.132	0.591
35	ORO‐F1‐38	4 December 2024	39	145	37.4	6	6.7	21.2	IV/V (silver)	0.170	0.567
36	ORO‐F1‐39	4 December 2024	39.4	130	41.3	5.1	5.5	27.3	IV/V (silver)	0.128	0.661
37	ORO‐F1‐40	4 December 2024	40.1	145	44.5	5.8	5.8	23.8	IV/V (silver)	0.130	0.535
38	ORO‐F1‐41	4 December 2024	47	185	46.2	6.5	6.7	22.4	IV/V (silver)	0.143	0.485
39	ORO‐F1‐42	4 December 2024	40.8	125	42.9	5.2	6.7	28.4	IV/V (silver)	0.139	0.662
40	ORO‐F1‐43	4 December 2024	49.1	225	51.1	3.7	4.6	25.2	IV/V (silver)	0.081	0.493
41	ORO‐F1‐44	4 December 2024	42.5	165	46.7	6.5	6.9	21.9	IV/V (silver)	0.143	0.469
42	ORO‐F1‐45	4 December 2024	38.2	145	41.2	6.4	6.1	24	IV/V (silver)	0.152	0.583
43	ORO‐F1‐46	4 December 2024	46	175	47.2	5.8	6.7	24.9	IV/V (silver)	0.132	0.528
44	ORO‐F1‐47	4 December 2024	47	245	50.9	5.3	6.3	26.5	IV/V (silver)	0.114	0.521
45	ORO‐F1‐48	4 December 2024	39.9	160	38.4	6.4	6.9	21.6	IV/V (silver)	0.173	0.563
46	ORO‐F1‐49	4 December 2024	51.1	245	57.5	6.1	7.5	34.8	IV/V (silver)	0.118	0.605
47	ORO‐F1‐50	4 December 2024	42.2	190	50.1	6.4	6.9	24.3	IV/V (silver)	0.133	0.485
48	ORO‐F1‐51	4 December 2024	40.5	150	41.8	5.9	7	22.2	IV/V (silver)	0.154	0.531
49	ORO‐F1‐52	4 December 2024	50.5	260	65.9	6.8	7.1	26.3	IV/V (silver)	0.105	0.399
50	ORO‐F1‐53	4 December 2024	45.7	210	50.6	6	6.1	29.1	IV/V (silver)	0.120	0.575
51	ORO‐F1‐54	4 December 2024	44.7	225	48.9	5.8	6.7	26	IV/V (silver)	0.128	0.532
52	ORO‐F1‐55	4 December 2024	42.3	190	47.8	6.1	6.2	23.8	IV/V (silver)	0.129	0.498
53	ORO‐F1‐56	4 December 2024	48.6	240	52.3	5.2	6.1	28.8	IV/V (silver)	0.108	0.551
54	C6_R1_E02	25 February 2025	37	110	47.3	6.4	5.9	27.08	IV/V (silver)	0.130	0.573
55	C6_R1_E08	25 February 2025	37.5	110	46.6	6.3	5.3	23.6	IV/V (silver)	0.124	0.506
56	C6_R1_E15	25 February 2025	35	90	41.1	5.8	5.5	22.5	IV/V (silver)	0.137	0.547
57	C9BR1_E01	27 February 2025	52	322	60.5	6.02	6.3	29.5	IV/V (silver)	0.102	0.488
58	C9BR1_E03	27 February 2025	56	355	66.1	6.8	8.06	37.3	IV/V (silver)	0.112	0.564

*Note*: The Durif Eye Index was calculated following Durif et al. (2005), using eye diameter and head length measurements to classify eels.

In the River Polis electric fishing, five eels were captured that exhibited morphometric characteristics consistent with advanced silvering (Stage IV/V), described by Durif et al. (2005) (Table 1). The Polis River is highly fragmented, with anthropogenic barriers restricting connectivity. The silver eels were captured across a number of sites (>10 km from shore), suggesting silvering occurs across the catchment. This demonstrates successful upstream migration by glass eels despite the presence of barriers, but also suggests that these same barriers, in conjunction with dry conditions, may hinder or delay downstream migration for some individuals. The combination of fully silvered morphology and late‐season capture raises the possibility of either delayed migration or the onset of de‐silvering, a phenomenon observed in eels held in captivity or blocked environments (Durif et al., 2009). These findings highlight the importance of considering river fragmentation and local environmental conditions when interpreting the dynamics of silvering. Taken together, these observations indicate that the fragmented nature of the Polis River catchment is likely preventing effective escapement of silver eels and thus is directly reducing the contribution to the eel spawning population of this system. Further investigation using physiological markers (e.g. hormone levels, gonadosomatic index) or tracking studies using telemetry could clarify whether these individuals represent postponed migrants or eels exhibiting physiological regression due to migratory disruption.

These results thus provide the first documented evidence of silver eel presence and attempted migration from inland waters of Cyprus, expanding the known geographic scope of silver eel migration and contributing to the broader understanding of eel life history and conservation needs. Although silver eel presence has been confirmed in Cyprus, evidencing the presence of all relevant eel life stages on the island, the wider significance of this finding for eel conservation more generally requires further investigation. In particular, the relative contribution of Cypriot eel populations to the overall Mediterranean spawning stock is currently unknown, but the confirmation of silver eel escapement from the island demonstrates that this now warrants focused investigation. In the short term, this finding of relatively large numbers of silver eels attempting to migrate to the sea should be used in two ways. Firstly, despite Natura 2000 status, Oroklini Lake was disconnected from the sea due to the structural design of the lake sluice and a road construction downstream of the sampling area also created an additional obstruction to migration. Thus, reconnecting this system to the sea to allow silver eel escapement is urgently needed. Secondly, the exemption of Cyprus from EMPs under EC No 1100/2007 due to the absence of substantial evidence for significant eel populations or migration in its freshwater habitats can now be challenged. This is considered the most important implication of these results at present, with recommendations that the results here are built on to generate further data on eel presence and abundance on the island so that silver eel escapement can subsequently be assessed in relation to eel biomass and survival in the presence and absence of anthropogenic pressures. This will then ensure the robust development of an EMP for Cyprus.

## AUTHOR CONTRIBUTIONS

S.M. conducted data analysis and prepared the manuscript. S.M., N.P.G., R.M.W., D.A. and M.I.V. carried out fieldwork and data collection. J.R.B. contributed to study design, data interpretation and manuscript revision. All authors approved the final manuscript.

## CONFLICT OF INTEREST STATEMENT

There are no conflicts of interest.

## FUNDING INFORMATION

This research was supported through match funding provided by the Environment Agency and Bournemouth University 13373.

## Data Availability

The data that support the findings of this study are available on request from the corresponding author. The data are not publicly available due to privacy or ethical restrictions.
